# Digital skills of health care professionals in cancer care: A systematic review

**DOI:** 10.1177/20552076241240907

**Published:** 2024-03-24

**Authors:** Tuominen Leena, Poraharju Jenna, Carrion Carme, Lehtiö Leeni, Leino-Kilpi Helena, Moretó Sònia, Stolt Minna, Sulosaari Virpi, Virtanen Heli

**Affiliations:** 1 Department of Nursing Science, 8058 University of Turku, Turku, Finland; 2 Comprehensive Cancer Center, Helsinki University Hospital, Helsinki, Finland; 3 3836 Intensive Care Unit, Helsinki University Hospital, Helsinki, Finland; 4 Research of Faculty of Health Sciences Studies, 16766 Open University of Catalonia (Universitat Oberta de Catalunya, UOC), Barcelona, Spain; 5 Turku University Library, University of Turku, Turku, Finland; 6 60652 Turku University Hospital, Turku, Finland; 7 16766 Open University of Catalonia (Universitat Oberta de Catalunya, UOC), Barcelona, Spain; 8 637679 Satakunta Wellbeing Services Country, Pori, Finland; 9 8056 Turku University of Applied Sciences, Health and Well-being, Master School, Advancing Supportive Cancer and Palliative Care (CARE)—Research Group, European Oncology Nursing Society, Turku, Finland

**Keywords:** Cancer care, digital skills, digital healthcare, health care professionals, oncology

## Abstract

**Background:**

The digital transformation of healthcare enables new ways of working in cancer care directing attention on the digital skills of healthcare professionals. This systematic review aims to identify existing evidence about digital skills among health care professionals in cancer care to identify the needs for future education and research.

**Methods:**

Database searches were conducted in PubMed, CINAHL, Web of Science, Scopus, Cochrane and ERIC to identify studies until March 2023. The inclusion criteria were digital skills of health care professionals in cancer care as described by themselves, other health care professionals, patients or significant others. The CASP tool was used for quality assessment of the studies. Data was analysed following inductive content analysis.

**Results:**

The search produced 4563 records, of which 24 studies were included (12 qualitative, 10 quantitative, 1 mixed methods design and 1 strategy paper). Four main categories were identified describing HCPs’ required skills, existing skills and development areas of digital skills in cancer care: Skills for information technology, Skills for ethical practice, Skills for creating a human-oriented relationship and Skills for digital education and support. In development areas, one more main category, Skills for implementing digital health, was identified.

**Conclusion:**

The digital skills of health care professionals in cancer care are multifaceted and fundamental for quality cancer care. The skills need to be assessed to provide education based on actual learning needs. The review findings can be used for education and research in this field.

## Introduction

In recent years, cancer care has been increasingly complemented by digital technology,^1,2^ including monitoring symptoms and facilitating adherence to treatment and care, promoting healthy lifestyles and user engagement with digital cancer services.^3^ The evidence supports the comparability of cancer care services produced digitally and in person in terms of patient satisfaction and effectiveness.^1^ Although the research evidence on digitally produced supportive cancer care is limited, improvements have been achieved in health-related outcomes.^2^ Successful implementation of digital technologies can significantly improve healthcare delivery in the European Region,^4^ but full exploitation of the potential of digital cancer care services requires advanced digital skills.^5–7^

There are few definitions of digital skills, and skills can be seen as an attribute to digital competence.^8,9^ The overlap of the two concept definitions makes it challenging to describe digital skills. Skills in general means the ability to apply and use knowledge to complete tasks and solve problems.^10^ Digital skills refer to the ability to access, manage, understand, integrate, communicate, evaluate and create information safely and appropriately. Furthermore, domains such as device and software operation, problem-solving and career-related competencies have been recognised in digital skills framework.^11^ This review focuses on digital skills defined as actions that HCPs take to support the health and resources of people with cancer to provide quality digital cancer care services. Competence is a broader concept encompassing skills and referring to the proven ability to use knowledge and skills as well as to personal, social and methodological abilities in professional development.^10^ It comprises (a) information and data literacy, communication and collaboration, digital content creation, and safety and problem-solving,^9^ (b) knowledge of digital technology, social and communication skills, and ethical considerations,^12^ and (c) self-assessed competence, knowledge and attitudes towards the use of digital technologies.^13^

Several recommendations related to digital skills have been reported. The emphasis has been on enhancing opportunities for digitalisation in cancer care^7^ and providing education, information and support digitally for people with cancer while maintaining confidentiality.^14^ Furthermore, HCPs need digital literacy, that is, abilities to use digital technologies to find, evaluate, create and communicate information,^12^ as well as apply the knowledge gained from electronic sources to address health problems.^15^ In their work, HCPs also collect, utilise and make decisions about digital health data. This requires skills for secure data handling and sharing whilst respecting the rights of individuals.^16^ The Code of Conduct for mobile health apps focusing on privacy and consent aims to increase trust in mobile health apps.^17^

The challenges in digital skills in cancer care have focused especially on developing a trusting relationship and providing person-centred care in a humane way.^18^ Person-centred care requires identifying patients’ willingness to use digital technology, evaluating patients’ digital capabilities and incorporating patients’ needs into digital cancer care services. However, how to conduct patient education in digital environments needs clarification.^19^ Challenges have also been related to physical examination and assessment of patients via digital tools^1^ as well as ethical practice, such as respecting patients’ privacy and confidentiality during the digital appointment.^20^ Recent studies assessing HCPs’ digital skills indicated that majority of participants (*n* = 803) demonstrated basic level of digital competence,^8^ whereas in low-income countries (*n* = 167) the level was relatively low.^21^ There is a research gap in assessing HCPs’ current digital skills in cancer care, and no previous literature review has been reported on the subject so far.

The purpose of this literature review is to identify existing evidence of digital skills among HCPs in cancer care to identify needs for future education and research. The review was guided by the following research questions:

1. What are the digital skills of HCPs in cancer care?
2. How should the digital skills of HCPs be developed in cancer care?

## Methods

This study adheres to the Preferred Reporting Items for Systematic reviews and Meta-Analyses (PRISMA)^22^ and is registered with PROSPERO (CRD42023413979).

### Eligibility criteria

Inclusion and exclusion criteria for the review are described in Table 1. We accepted studies published from the establishment of the databases until March 2023.

**Table 1.** Eligibility criteria of included studies.

Inclusion criteria	Exclusion criteria
Health care professionals’ digital skills described by themselves, other health care professionals, patients or significant others	Other than health care professionals’ (patients’, family members’, informal caregivers’, students’) digital skills described
Description is focused on health care professionals’ digital skills in aims, methods or results of the study	Description is not focused on health care professionals’ digital skills (but rather on application, equipment, technology or educational program) in aims, methods or results of the study
Research studies, proceedings, strategy papers, theoretical models	Protocol articles, reviews, posters, book chapters, editorials, letters
Cancer care setting	Other setting than cancer care; for example, digital learning

### Information sources

A systematic literature search was conducted in collaboration with the researchers and Information Specialist to six databases to identify eligible studies: PubMed, CINAHL, Web of Science, Scopus, Cochrane and ERIC. Additionally, references cited in the study reports included in the systematic review were screened to identify additional studies.

### Search strategy

The literature search was conducted with three main concepts and their synonyms: digitalisation, skills and search were limited to peer reviewed journals and English language (Supplementary File 1). The search strategy was validated by test searches and discussion in the research group.

### Selection process

Records were imported to Covidence software that assisted with the screening process and duplicates were removed. First, the records were screened independently by two researchers of the research team (CC, HLK, HV, JP, LT, TF or VS) based on title and abstract according to the eligibility criteria (Table 1) and conflicts were resolved by a third reviewer. Second, full texts were screened independently by two researchers according to the eligibility criteria and conflicts were resolved by a third reviewer of the research team (AB, CC, FSR, HLK, HV, JP, LT or SM).

### Data collection process

Data was collected as follows: (a) reference, year, country, (b) purpose, (c) method, (d) theoretical approach, variables or focus of the research, (e) participants, (f) data collection, (g) analysis method, (h) findings related to digital skills and (j) conclusions related to digital skills. Data extraction was conducted independently by two researchers (LT and JP) and disagreements were resolved in consultation with a third person of the research team (HV or HLK).^23^

### Data analysis

Purpose of the analysis was to describe HCPs’ digital skills. Data comprised both qualitative and quantitative studies, and based on that, inductive content analysis was used.^24,25^ The coding of the content focused mainly on describing the manifest content of the literature. Since the studies did not actually assess HCP's digital skills, the analysis focused on the descriptions of digital skills in the results, discussion or conclusion sections of the studies. The analysis included five steps for each research question.^25^ First, the researchers familiarised themselves with the data by reading the texts. Second, data was divided into meaning units and abstracted into codes related to digital skills. Third, the content and context of the codes were compared with each other, and corresponding codes were grouped into sub-categories. Fourth, the sub-categories were compared with each other, and based on similarities and differences, main categories were formed. Main categories were named according to their content.^24,25^ Finally, the main categories were compared with each other, and their names were refined. During the whole abstraction process, construction of sub- and main categories and decisions was made in consensus among the research team (LT, JP, HV and HLK). In reporting, study characteristics and findings were tabulated, and representative quotes were used for supporting the credibility of the findings.^24^

### Quality appraisal of the studies

Articles were assessed independently by two researchers (SM and CC). Disagreements were discussed until consensus was reached. For qualitative studies, a 10-item CASP scale (0–10) for qualitative studies was used^26^ focusing on (a) validity of the study, (b) accuracy of the results and (c) transferability. For quantitative studies, a 12-item CASP scale (0–12) for cohort studies was used^27^ focusing on (a) validity of the study, (b) internal validity of the results and (c) external validity. Items 6A, 6B, 7 and 8 were not applicable for included cross-sectional studies, they were thus omitted. When there was a ‘No’ for questions 1 and/or 2, the quality assessment was not completed with follow-up questions.^31^ Quality appraisal was used to demonstrate the methodological quality of the studies as it affects the validity of the findings of the review.^27^

## Results

**Figure 1.**
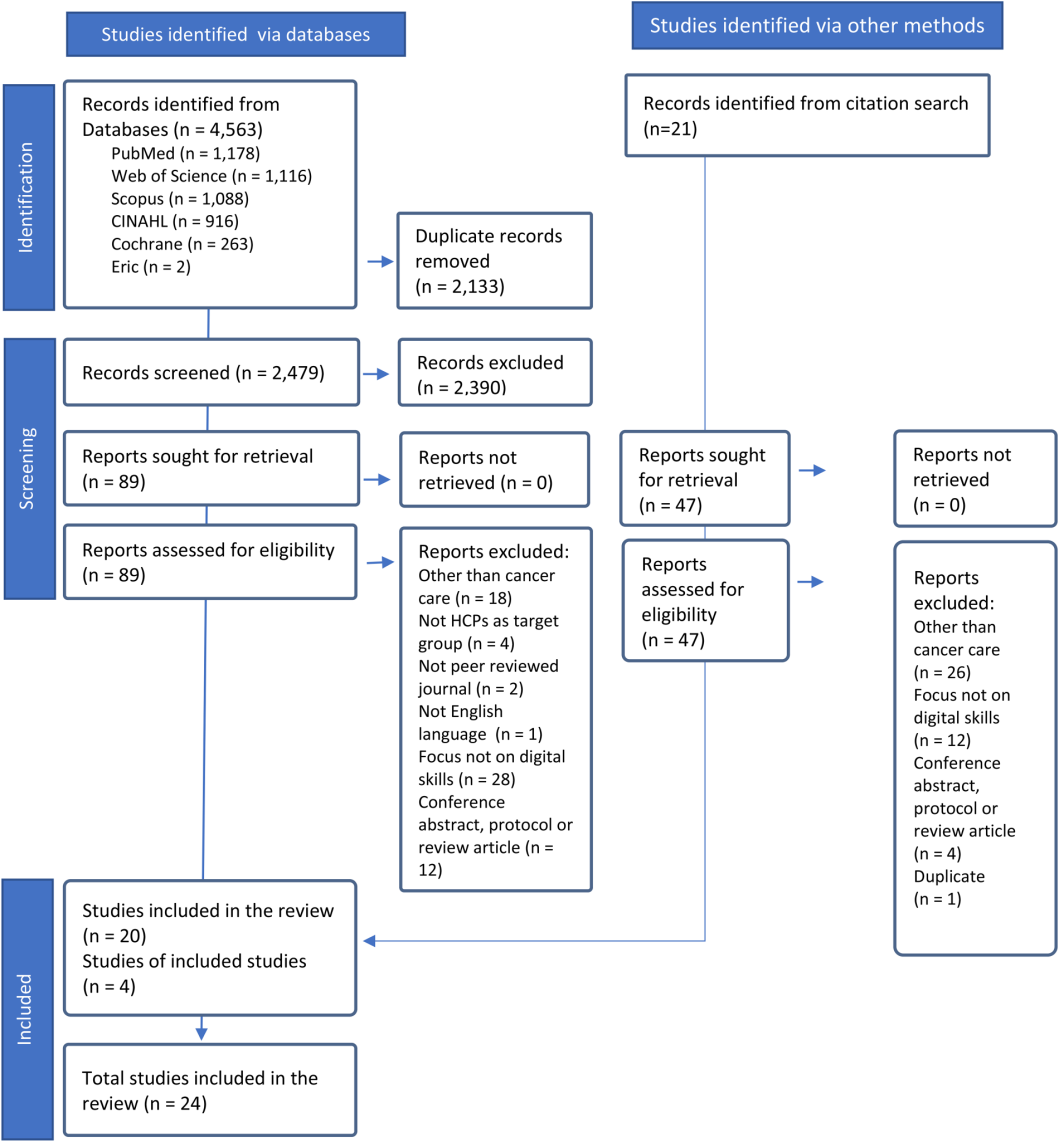
PRISMA flow chart for the study search and selection process.^22^

Of the 4563 records identified a total of 24 studies met the inclusion criteria (Figure 1).

### Study characteristics

The included studies were published during the last 18 years (2005–2023). Of those, 67% were published during the last five years. The studies were conducted in USA,^28–36^ Canada,^37–40^ Australia,^41–44^ England,^45,46^ Netherlands,^47,48^ Denmark,^49^ Rwanda^50^ and Turkey.^51^ Designs of the studies were qualitative (*n* = 12, 50%),^28,31,32,34,35,38,40,41,43,48–50^ quantitative (*n* = 10, 42%),^29,30,33,36,39,42,44,46,47,51^ mixed method (*n* = 1, 4%)^41^ and a strategy paper (*n* = 1, 4%).^45^ Data was collected primarily by interviews (*n* = 10, 42%),^31,32,34,35,37,38,41,43,49,50^ or by surveys (*n* = 7, 30%).^29,30,33,36,42,47,51^ The participants were HCPs in different cancer care settings: nurses (*n* = 4, 8%),^31,33,37,39^ medical oncology providers (*n* = 1, 4%),^32^ radiation oncology nurses (*n* = 1, 4%),^28^ radiation oncologists (*n* = 2, 8%),^30,51^ surgical learners (*n* = 1, 4%),^29^ psychosocial cancer care providers (*n* = 2, 8%)^36,40^ and trainees and fellows in radiology/radiation oncology (*n* = 1, 4%).^42^ In six studies (25%), the participants represented several professions.^32,46–48,50^ In two studies, the participants were people with cancer (*n* = 2, 8%),^38,49^ and in two studies, both nurses and people with cancer (*n* = 2, 8%).^35,44^ In two studies, care givers were included with people with cancer and/or HCPs (*n* = 2, 8%).^34,41^ One study had a broad sample of consumers and other stakeholders (*n* = 1, 4%)^43^ (Table 2). Skills were most often reported in connection with using digital technology or as HCPs’ concerns related to the skills. None of the studies assessed the level of these skills.

**Table 2.** Study characteristics (*n* = 24).

Authors Country, year	Purpose	Method (a) Design, (b) data collection and (c) data analysis	Population (a) Setting, (b) sample and (c) gender	Results related to required and existing skills and development areas in digital skills of HCPs	Quality (CASP)
**Adames et al.^28^** **USA** **2023**	To examine the impact of telehealth changes on nursing care in the radiation oncology setting at a Comprehensive Cancer Care Centre during the COVID-19 pandemic.	(a) Qualitative design (b) Semi-structured interviews (focus group) in March 2021 (c) Thematic analysis	(a) New York City / outside New York City (b) Nurses (*n* = 18) (c) Not reported	Nurses were challenged to learn and operate various digital platforms without training. Nurses were unable to perform physical assessment over the phone. Nurses found alternative ways to obtain important assessment information, e.g., by uploading pictures. Nurses experienced distractions during the telehealth appointment. Telehealth evoked issues related to privacy and confidentially. Nurses identified a need for institutions to update their telehealth policies.	7/10
**Banerjee et al.^45^** **England** **2022**	To enhance clinician readiness to provide high-quality care to people with cancer the paper presents a tele-oncology communication guide that utilises the best practices of an adapted communication skills training.	A strategy paper	Not applicable	Comskil TeleOnc Guide for best practices for communication skills in virtual cancer care lists four components of HCP/patient/ family communication: (a) communication goal, (b) communication strategies, (c) communication skills and (d) process tasks. Skills unique to telehealth are ensuring privacy, technology check and technology back-up plan. The suggested approaches to achieve communication goals include forming a strong clinician-patient relationship, building rapport, setting the agenda, demonstrating empathy in response to emotions, delivering the information and concluding the tele-oncology visit effectively.	N/A
**Borycki et al.^37^** **Canada** **2019**	To explore the impact of integrating electronic symptom management guidelines and electronic health records upon oncology, telehealth nursing practice and to examine the use of eSMGs by telehealth nurses in oncology settings.	(a) Mixed method with qualitative emphasis (b) Post-clinical simulation interviews (c) Content analysis	(a) Telehealth nursing settings at a large multi-site oncology treatment organisation. (b) Oncology nurses (*n* = 10) (c) 100% female	Some nurses proposed organisations to create methods for informing nurses about updates to the guidelines, aiming to improve their awareness of information.	7/10
**Bødtcher et al.^49^** **Denmark** **2022**	To investigate how people with cancer experienced the COVID-19 pandemic and how they perceived the change from in-person consultations to telephone consultations in an oncology outpatient clinic	(a) Qualitative design (b) Semi-structured individual interviews (c) Thematic analysis	(a) The Department of Clinical Oncology and Palliative Care, Zealand University Hospital, Denmark. (b) People with cancer (*n* = 15) (c) Equal gender distribution	HCPs’ actions affected patient satisfaction during telehealth. Patients were satisfied when the physicians called on time, allocated enough time for the consultation, provided sufficient and relevant information, and addressed concerns effectively. Patients requested information to be prepared for consultations and encouragement to write down questions during the call. Loss of information and nuances during telehealth, physicians’ language and communicative skills affected patientś experience. Patients suggested telephone consultations for follow-up, uncomplicated diseases, information that is short or practical, positive test results or with a familiar physician.	6/10
**Damico et al.^30^** **USA** **2022**	(a) To assess patterns of telemedicine usage among radiation oncology physicians before and after the COVID-19 pandemic. (b) To describe satisfaction and perceived value of telemedicine, barriers to telemedicine use.	(a) Quantitative design (b) Electronic survey, distributed internationally, June to October 2020 (c) Descriptive statistics	(a) 78.0% from USA (b) Radiation oncologist (*n* = 232) (c) 63.8% male, 31.8%female	Perceived value: Radiation oncologists were able to express themselves effectively using telemedicine, 21.3% agreed that appointments in person are more effective, 6.0% of users had concerns regarding HIPAA and/or patient privacy. Perceived barriers to telemedicine use were inability to perform physical examination and establish/maintain rapport with patients equally well using telemedicine compared to in-person appointments.	6/10
**Dickerson et al.^31^** **USA** **2005**	To understand the experiences of oncology nurses who use the Internet in their daily practice when their patients use the Internet for cancer care.	(a) Qualitative design (b) Informal interviews (c) Hermeneutic process	(a) Cancer Centre (6), clinics (6), community hospitals (4), veterans’ hospitals (3), Internet companies (4) (b) Oncology nurses (*n* = 20) (c) 95% female, 5% male	The Internet changed the nurse-patient relationship to a partnership where both works to achieve a common goal. Nurses’ role changed from educator to consultant. Nurses need to know how to search and assess information on the Internet. Nurses facilitated patients’ information searches by individualising search information, determining its trustworthiness and credibility and correcting misinformation. Skills required to practice in the Internet era: computer proficiency, evaluation of information, e-mail communications with patients and content development for online sources.	4/10
**Emond et al.^47^** **Netherlands** **2013**	Investigates HCPs’ opinions, cognitions and behaviour regarding referring cancer patients to Internet-based information.	(a) Quantitative design (b) Explorative survey (c) Descriptive statistics	(a) HCPs involved in education of cancer patients in all hospitals in the province of Limburg (b) Health professionals (*n* = 130); 32% medical specialists, 46% Oncology nurses (c) Medical specialists: 69.7% male, 30.3% female. Oncology nurses: 87% female, 13.2% male	HCPs were unsure about their own Internet referral capacity. 20% of HCPs often or always refer patients to cancer-related websites for more information. 32% of HCPs indicated that reminders would help them to refer more patients to the websites. On average, HCPs judged themselves to be skilled in the use of computers and the Internet (1 = inadequately skilled to 4 = very skilled; *M* = 3.09; *SD* = 0.71).	7/10
**Gotlib Conn et al.^38^** **Canada** **2021**	To examine (a) patient-perceived risks related to COVID-19 and experiences with outpatient cancer treatment and (b) patient-perceived and experienced benefits and drawbacks of virtual cancer care.	(a) Qualitative design (b) Semi-structured interviews (focus group, individual), June to August 2020 (c) Thematic analysis	(a) The Odette Cancer Centre at Sunnybrook Health Sciences Centre in Toronto. (b) Patients (*n* = 24) (c) 54.2% female, 45.8% male	Benefits of virtual cancer care: physicians called at the agreed time and allocated sufficient time for telehealth appointment, HCPs were patient and respectful for scheduled appointments. Drawbacks of virtual cancer care: The possibility to conduct full physical assessment on virtual care, a tendency to forget questions more often during virtual visits compared to in-person ones. It is important to consider patients’ preferences due to the diverse range of comfort levels with technology, potential language or communication barriers and varying desires for either virtual or in-person interactions with their physicians when considering virtual appointments.	6/10
**Handley et al.^32^** **USA** **2022**	To identify medical oncology providers’ perceptions of telehealth video visits as influenced by the COVID-19 pandemic and to examine HCPs’ willingness to engage in video visits and the barriers that continue to limit successful telehealth use for medical oncology care.	(a) A prospective qualitative design (b) Semi-structured interviews, survey (c) Content analysis	(a) Sidney Kimmel Cancer Center at Thomas Jefferson University, a National Cancer Institute designated cancer centre in Philadelphia. (b) Medical oncology providers (*n* = 25); 18 physicians, 7 advanced practice providers. (c) 52% female, 48% male. 76% white	Barriers to telehealth visits: It is important to have clear communication with patients regarding an individual patient's perspective of telehealth acceptability and tailor its use to the patient's preference. Barriers to continuing telehealth use: difficulties to connect with patients, concerns regarding ability to forge or maintain a robust provider-patient relationship via telehealth visits, limited physical examination capabilities via telehealth resulting in faulty diagnoses or inappropriate treatment.	9/10
**Hughes et al.^33^** **USA** **2014**	To address the need to improve computer proficiency of registered nurse (RN) and improve IT skills.	(a) Quantitative design (b) Pre-and post-intervention surveys (c) Descriptive statistics	(a) A nurse-led, educational intervention in ambulatory genitourinary oncology unit (b) RN (*n* = 53); pre-intervention (*n* = 32), post-intervention (*n* = 14)	Pre-intervention survey identified a need to improve nurses computer proficiency. Post-intervention survey found that 78% of the nurses improved work-based computer skills and 78% improved comfort level. The intervention improved RN proficiency in finding items and overall computer skills and comfort level.	2/9
**Jørgensen et al.^34^** **USA** **2021**	To explore patients’ and caregivers’ experience of calling an oncological emergency telephone.	(a) Qualitative design (b) Semi-structured interviews, September 2016 to May 2017 (c) Content analysis	(a) Department of Oncology, Danish university hospital. (b) 8 Patients and 4 caregivers (*n* = 12) (c) Patients: 75% female, 25% male. Caregivers 75% female, 25% male	HCPs’ communication style during the telehealth affects patients’ experiences. If HCPs’ tone and attitude lacks empathy, patients may feel unwelcome to share their symptoms. Understating symptoms makes patients and caregivers feel rejected and undermines trust in both the HCPs and the healthcare system. When HCPs use caring communication, show professional competence and engage supportively, it fosters trust, build relationships and enhance the sense of being cared for.	8/10
**Karera et al.^50^** **Uganda** **2022**	(a) To understand HCPs’ views and perceptions of the use of mHealth as part of palliative care delivery. (b) To explore HCPs’ perceived role in mHealth in palliative cancer care, the challenges associated with its use and how it might support the advancement of palliative cancer care	(a) Qualitative design, supra-analysis (b) Semi-structured interviews (c) Thematic analysis	(a) HCPs (*n* = 20); 10 nurses, 6 physicians, 2 social workers, a counsellor and clinical officer. (b) 75% female, 25% male	Challenges in use of mHealth: Misunderstandings during mHealth communication leads to misinterpretations in patients, HCPs and care givers; confidentiality; patients’ information is accessed without their knowledge or consent in instances where mobile phones are shared between households. mHealth mobile applications should be password- protected.	4/10
**Kemp et al.^43^** **Australia** **2021**	To examine consumer and other stakeholder views on implementation of digital health approaches in cancer care in Australia.	(a) Qualitative design (b) Semi-structured interviews (individual, paired, focus group), June 2018–January 2019 (c) Thematic analysis	(a) Stakeholders (*n* = 51); 14 people with a history of cancer /cancer caregiving, 9 HCPs, 6 researchers, 6 representatives of non-government cancer service providers, 6 digital health technology developers and 10 representatives in state or federal government health or digital health policy positions.	On implementation of digital health, addressing digital health literacy involves tailoring digital health approaches to individual requirements, especially for older individuals, socioeconomically disadvantaged populations, and those with lower digital proficiency. Education is essential for enhancing user skills in utilising digital technology increasing awareness of available services and guiding informed choices by consumers and healthcare professionals regarding safe and suitable digital health resources.	5/10
**Koppel et al.^35^** **USA** **2022**	To explore the experiences of nurses and patients participating in oncology telehealth VCVs, specifically in relation to the cultivation of rapport.	(a) Qualitative descriptive study (b) Semi-structured interviews, October 2021– March 2022. (c) Content analysis	(a) Magnet- and NCI-designated Comprehensive Cancer Centre in a north-eastern metropolitan area of the United States. (b) 10 Patients and 12 nurses (*n* = 22) (c) Patients: 70% women, 30% men Nurses: 84.4% female, 15.6% male	Patients’ experiences of VCVs: Appreciated nurses asking personal questions and remembering personal details about patients. Appreciated nurses sharing things about themselves. Nurses’ experiences of VCVs: Patient's experience with videoconferencing technology might be a barrier for cultivation of rapport, mixed impressions about telehealth's impact on rapport building, important to determine when virtual appointment is feasible for patient with cancer*,* patients have varying degrees of comfort with technology, potential language or communication barriers, patients might prefer face-to-face appointments.	8/10
**Korkmaz et al.^51^** **Turkey** **2023**	To investigate the perception of radiation oncologists about artificial intelligence (AI), their current use of AI in clinical practice, and their expectations, concerns and wishes in terms of the future of radiation oncology (RO) in the era of AI.	(a) Quantitative design (b) Cross-sectional electronic survey July 2021– September 2021 (c) Descriptive statistics	(a) Turkish Society of Radiation Oncology, participants mainly practiced in university (50%) (b) Radiation oncologists (*n* = 108); RO specialists 39%, professors 26%, associated professors 18%, attendees 8%.	Radiation oncologists need regular training and education sessions about fundamentals of AI and its latest developments, clinical validation of AI applications before being introduced into clinical practice.	3/9
**Macartney et al.^39^** **Canada** **2012**	To identify the most prevalent and challenging symptom management issues for oncology nurses providing remote symptom support.	(a) Quantitative design (b) Online survey (c) Descriptive statistics, content analysis	(a) Chemotherapy administration 55%, supportive care 37%, palliative care 32% and radiation therapy 24%. (b) Oncology nurses (*n* = 368);	Of the nurses, 21% expressed a challenge in obtaining relevant patient information during remote symptom assessments due to lack of visual patient assessment, communication complexities and doubts about the accuracy of patient medical history and medication details.	5/9
**Maraiki et al.^46^** **England** **2018**	To evaluate the HCPs’ knowledge of the current institutional e-resources in an adult oncology setting.	(a) Quantitative design, (b) Survey (c) Descriptive statistics	(a) An adult oncology setting. Staff from in-patient, outpatient and combined practice settings. (b) Practitioners (*n* = 141); 66 physicians, 200 nurses, 27 pharmacists.	Less than 50% of the HCPs were aware of, able to access or frequently utilised institution specific e-resources. 75% of the HCPs had never utilised institution-specific e-resources. Sending alerts/emails for newly uploaded material would encourage accessing and utilising e-resources. The low overall awareness for the institutional e-resources highlights the need for continuous and structured training.	5/9
**Mooi et al.^41^** **Austalia** **2012**	To assess the satisfaction level and perspectives of Indigenous patients, their families and health care workers (HWs) working with Indigenous patients on videoconference (VC) and the tele oncology service.	(a) Qualitative design (b) Semi-structured interviews, patients participated in VC 2007–2011 (c) Descriptive statistics, thematic analysis	(a) Tertiary referral centre (Townsville Cancer Centre) and various rural and remote towns in Queensland. (b) Nine patients, 2 family members and 6 health workers (*n* = 17) (c) Patients: 66% female, 34% male.	HWs perceived that a good rapport was established between the patient and the specialist; most had no technological problems. Resistance to new technology may limit the adoption of VC among unexperienced users. Most had no medicolegal concerns with VC. Differential responses from patients and HWs about the role of HWs in VC highlights the issues of informed consent and patient confidentiality during the VC. All HWs participating in VC and the tele oncology service should undergo formal competency training in communication skills, basic operational skills for VC equipment and cultural awareness. VC clinical documentation needs to be recorded.	4/10
**Rivet et al.^29^ 2023** **USA**	To explore the value and utility of providing a simulation-based telehealth training programme to teach surgical residents and faculty best practices for disclosing difficult news.	(a) Quantitative design, a single-institution, single arm, unblinded, feasibility study (b) Electronic survey (c) Descriptive statistics	(a) A tertiary care center located in Central Virginia (b) Interns (11%), residents (46%) and faculty (39%) from General, Neuro, Paediatric, Plastics, Oncology, Urology and Vascular surgical specialties (*n* = 28). (c) 61% male, 39% female	When delivering difficult news remotely in ‘Prep-SPIKES for Delivering Bad News in Face-to-Face vs Telehealth Media’ telehealth simulation, participants had difficulties in conveying empathy due to limited visibility of facial expressions, the absence of physical touch or comfort offerings and potential awkwardness caused by audio or video disruptions. Distinct strategies are needed when delivering difficult news via video. HCPs are advised to anticipate and prepare for technical problems while delivering challenging news. Of the responses, 57% were positive for the overall quality of telehealth interactions. 68% felt at ease communicating through telehealth. 75% believed they could convey their emotional reactions through video-mediated communication.	3/9
**Scheetz et al.^42^** **New Zealand, Australia** **2021**	To ascertain the current use, understanding and perceptions of AI.	(a) Quantitative design (b) Online survey, June– August 2019 (c) Statistical analysis	(a) Metropolitan and rural areas (b) Fellows and trainees (*n* = 632); 305 ophthalmology, 230 radiology/radiation oncology, 97 dermatology.	There is a need for specialised colleges and representative organisations to take the lead in educating AI. The importance of cooperation in creating, deploying and controlling AI technologies in the healthcare sector was highlighted.	7/10
**Schnur & Montgomery 2012^36^** **USA**	To understand the state of e-Counselling practice and training in cancer care. To conduct an online professional training needs assessment with psychosocial cancer care providers.	(a) Quantitative design, cross-sectional (b) Online survey, March–April 2011. (c) Statistical analysis	(a) NCI-designated CCC 40%, general hospital with a cancer clinic 29%, academic cancer centre 20%, private clinic 6%, group oncology practice 5%. (b) Psychosocial cancer care providers (*n* = 120); psychologists 62%, nurses 10%, psychiatrists 2%	88% of the participants felt they did not currently have the skills to effectively conduct e-counselling.	3/9
**Shaw et al.^44^** **2013 Australia**	To describe communication behaviours within supportive care telephone calls in two contexts: (a) a telephone outreach intervention and (b) cancer helpline calls, to identify potential areas for further training to identify potential targets for training in health professional-patient telephone-based communication.	(a) Quantitative cross-sectional retrospective design (b) Pre-recorded CONNECT and helpline calls (c) Content analysis	(a) A randomised trial of telephone-based care coordination and supportive care intervention for colorectal cancer patients and Australian telephone information and support helpline operated by the Cancer Councils of Queensland and NSW (b) Existing recorded telephone calls with patient diagnosed with cancer (*n* = 50) (c) Patients: 64% female, 36% male.	The most frequent communication behaviours observed during telephone consultations were closed questions, paying attention to verbal cues and providing information or advice. Training in communication skills may improve telephone-based supportive care delivery. Those delivering supportive care via telephone need training to identify patients’ underlying emotional needs. Communication training programmes need to focus on developing interview techniques with open-ended questioning and empathic statements to facilitate patient discussion of concerns. They need to focus on responding verbally to patient emotional cues and verbally expressing empathy. Checking understanding accounted for less than 10% of total utterances. Paraphrasing and checking patient understanding should be emphasised in training. The lack of verbal cues requires more explicit clarification of patient understanding of the information provided.	5/9
**Stephen et al.^40^** **Canada** **2011**	To describe the experiences of expert psycho-oncology counsellors of facilitating online support groups (OSGs) and identify the important elements of their learning experience that led to engagement.	(a) Qualitative longitudinal design using interpretive descriptive methodology (b) Panel discussion (c) Content analysis	(a) The Wellness Community (b) Senior psychosocial oncology counsellors (*n* = 6); 2 Psychologists, 3 social workers and clinical counsellor, from five different cancer centres (c) 83.3% female, 16.7% male	The counsellors started adapting and using group facilitation skills in online support groups for cancer patients: Setting norms, establishing oneself as the leader of the group, building group cohesion by encouraging full participation, pacing discussion so everyone is heard, creating emotional immediacy and use of emotional self-disclosure, managing intense or potentially harmful emotions, learning to use the online tools to enhance group objectives.	4/10
**Stuij et al.^48^** **Netherlands** **2018**	(a) To uncover the learning needs of oncological healthcare providers related to information provision. (b) To explore training preferences of oncological healthcare providers in the context of clinical practice with respect to a new digital training tool.	(a) Qualitative design, contextual inquiry. (b) Interviews (focus group, individual), December 2016 to March 2017 (c) Content analysis	(a) Four academic medical centres and 2 peripheral medical centres across the Netherlands. (b) Oncological health care practitioners (HCP) (*n* = 16); 8 haematologists, 2 radiotherapists and 6 clinical nurse specialists (c) 62.5% female, 37.5% male	Themes related to HCPs’ learning needs in digital information provision were (a) tailoring information according to patients’ situation and needs, (b) structuring information and (c) dealing with emotions.	8/10

### Quality appraisal results

In the CASP checklist for 13 qualitative studies (Supplementary File 2), most of the studies showed a clear statement of the aims of the research, the qualitative study design was adequate, the findings were clearly defined, data collection and analysis were adequately rigorous in nine of the studies, and nine of them were designed in an appropriate way to achieve the aims of the research. However, recruitment strategy was considered appropriate in only three studies, and study results were likewise defined as being transferred to different settings in only three studies.

In the CASP checklist for 11 cohort studies (Supplementary File 3), most of the studies clearly stated their research objective, but only four of the 11 studies recruited the study population according to the aims of the research, and most of them used a convenience sampling strategy. Due to a cross-sectional study design, none of the studies measured exposure prior to the outcome, considered potential cofounding variables nor had a long enough follow-up. All studies have good internal validity but none of them has enough external validity to implement the results into different settings.

### Digital skills of health care professionals in cancer care

The digital skills are analysed based on descriptions in the Results, Discussion and Conclusion sections of the studies. In the analysis, we identified three themes: required and existing skills and development areas in digital skills of HCPs. The required skills were those that were identified as skills that HCPs need to have to provide digital services. Existing skills were those that studies found HCPs to already have. Development areas were digital skills that, according to studies, still need improvement. In each of these themes, the following four main content categories were identified:

1. Skills for information technology
2. Skills for ethical practice
3. Skills for creating a human-oriented relationship
4. Skills for digital education and support

In addition, one main category, ‘Skills for implementing digital health’, was identified as development area of HCPs’ digital skills. (Table 3). Of the main categories, ‘Skills for information technology’ consists of four sub-categories, ‘Skills for ethical practice’ of three sub-categories, ‘Skills for Creating a human oriented relationship’ of four sub-categories and ‘Skills for digital patient education and support’ of six sub-categories. However, not all the above sub-categories appear in every question considered. In addition, ‘Skills for implementing digital health’ comprises two sub-categories. Next, digital skills identified as required, existing and development areas are presented in more details.

**Table 3.** Study findings related to digital skills.

Digital skills Main Sub-categories categories		Required skills	Existing skills	Development areas
**Information technology**	Using digital technologies	Find alternative ways to assess patients, Operate various digital platforms,^28^ Integrate digital technologies into nursing programmes,^31^ Access the Internet,^31^ use the Internet for searching and filtering online information, Develop the content, Functionality and design of online sources.^31^	Having work-based computer skills,^33^ Searching information from databases.^31^	Learning basic operational skills for digital equipment,^41^ Assessing and developing computer skills.^41^
	Preparing digital appointment	Technological skills,^45^ predicting technical problems,^29,35,45^ Respecting the schedule.^38,49^		
	Assessing digital health information		Assessing information sources and website quality,^31^ Filtering relevant information from the databases.^31^	Providing HCPs information about the content and reliability of cancer-related websites,^47^ Guiding HCPs’ choices as to appropriate and safe digital health resources and system.^43^
	Accessing digital resources			Assisting patients to navigate digital health,^43^ Adapting new technologies,^42,51^ Reminding HCPs about updated guidelines,^37^ Enhancing HCPs’ awareness of institutional e-resources^46^
**Ethical practice**	Protecting patients’ privacy	Ensure appropriate setting for digital appointment.^45^		Protecting patients’ privacy during digital meetings.^28,30^
	Ensuring confidentiality	Protect patients’ passwords.^50^		Ensuring confidential conversation,^28^ Accessing patients’ information with their consent,^50^ Participation of a third person in a digital meeting.^41^
	Ensuring patients’ consent	Consent to digital appointment,^41^ Consent to the presence of third parties,^41^ Ask permission for recording,^41^ Request in-person consultation		
**Creating a human oriented relationship**	Adapting a person-centred approach	Work with patients to create person-centred options,^35^ Know patients as persons,^35^ Tailor digital health approaches to suit individual needs,^32,43^ check patients’ preference,^45^ Take account patients’ language difficulties,^35^ hearing impairments^35^ and cultural background.^41^		
	Building rapport	Make introductions,^45^ Partnering with the patient to ensure the concerns are responded to,^45^ Communicate with patients in a caring way,^34^ Use personal touch.^35^	Adapting bedside manner during video- conferencing,^35^ Using a caring tone of voice and guidance.^34^	Developing skills to establish a relationship,^34^ Create an emotional connection with patients,^32^ Maintaining a rapport.^30^
	Delivering difficult news			Replacing physical touch,^29^ Responding to patients’ emotions,^44,48^ Dealing with patients emotional reactions,^48^ Responding verbally to patients’ emotional cues,^44^ Verbalising empathy,^44^ Legitimising patients’ concerns.^44^
**Digital patient education and support**	Providing e-counselling			Lacking skills for providing e-counselling in 2012.^36^
	Individualising education			Determining what type of meeting will best serve the patient for each care encounter,^35,38,43,49^ Tailoring information for patients’ characteristics.^48^
	HCP–patient interaction		e-mailing with patients,^31^ communicate with care and support,^34^ Expressing oneself affectively digitally,^29^ Expressing oneself effectively and affectively,^30^ Assessing patient reported health information online,^31^ Respecting the scheduled appointment times,^38^ Connecting at unscheduled times.^38^	Having the necessary communication skills,^41,49^ Keeping nuances during digital communication,^49^ Using different digital media in communication,^29^ Preserving the human component of interaction,^38^ Showing facial expressions,^29,44,48^ Being present with a caring tone of voice and guidance.^34^
	Providing information	Set a common agenda for appointment,^45^ Deliver the key information,^45^ End appointment by summarising the main points and review next steps,^45^ Information consulting.^31^		
	Obtaining necessary information			Receiving accurate information,^39^ Reading patients’ reaction to the information provided,^49^ Assessing patients’ condition remotely,^28,38^ Performing a physical examination,^30,32^ Documenting each digital appointment.^41^
	Ensuring understanding	Provide information in a structured manner,^45^ Encourage patients to participate, Use silence to help patients process information,^45^ Check patients’ understanding^45^		Supporting patients to be prepared for digital appointment,^49^ Presenting health information in a structured way,^48^ Managing information,^48^ Avoiding misinterpretations,^50^ Focusing on interview techniques with open-ended questions,^44^ Encouraging patients to ask questions,^38,49^ Checking patients’ understanding.^44^
	Providing support	Encourage expression of patients’ feelings,^45^ Respond empathically to patient's emotions, verbally acknowledging support,^45^ Acknowledge patient's emotional cues,^45^ Affect patient's emotional response.^45^	Being a facilitator in the online support group chat (OSGC), Acting as group leader, setting norms, pacing discussion, sing emotional self-disclosure, anaging intense emotions, Using online tools to enhance group objectives during the OSGC.^40^	
**Implementing digital health**	Creating a standard digital health policy			Updating institution practices on digital health,^28^ Defining factors that constitute a good digital appointment.^34,49^
	Implementing digital health guidelines			Ensuring the safety of digital technologies,^42,51^ Creating frameworks for the development, adoption and regulation of AI technologies,^42^ Complying with the law,^51^ Ensuring person-centred practice guidelines.^35^

### Required digital skills of health care professionals in cancer care

The required skills are based on descriptions by HCPs themselves,^28,29,31–33,41,50^ patients,^49^ or both HCPs and patients^34,35^ and as strategy outlines.^45^ The digital skills required of HCPs formed four main categories: skills for (a) information technology,^28,29,31,35,38,45,49^ (b) ethical practice,^32,41,45,50^ (c) human-oriented relationship^34,35,45^ and (d) digital patient education and support^45,49^ (Table 3).

Required skills for information technology comprise two sub-categories: using digital technologies^28,31^ and technological preparing of digital appointments.^29,35,38,45,49^ Using digital technologies includes skills to find alternative ways to assess patients and operating various digital platforms.^28^ It also includes skills to integrate digital technologies into clinical practice, access the Internet and search and filter online information. In addition, it includes developing the content, functionality and design of online sources.^31^ The following quotation expresses the requirements for using digital technologies:

The nurses need to know how to access the Web and how to search and separate good and bad sites.^31^

Preparing digital appointment includes respecting the schedule, such as calling the patient at the agreed time and allocating sufficient time for the appointment.^38,49^ It also includes technological skills such as technology check,^45^ setting the camera position and lighting for the digital appointment,^29^ as well as predicting technical problems^29,35,45^:

For FTF encounters, seating arrangements, chair height, and eye level can impact the dynamics of the interaction, while on video the emphasis should be on camera position, appropriate front lighting, and minimizing technical issues with plans for trouble-shooting.^29^

Required skills for ethical practice comprise two sub-categories: protecting patients’ privacy^45^ and ensuring confidentiality^50^ and patients’ consent.^41^ Protecting patients’ privacy includes ensuring the setting is appropriate for health information discussion.^45^ Confidentiality includes protecting patients’ passwords during digital appointment^50^:

To maintain patient confidentiality, which may be compromised using personal mobile phones, most participants suggested that the mobile applications should be password protected.^50^

Ensuring patients’ consent includes asking patients’ permission for a digital appointment. In addition, it includes ensuring patients’ consent to the presence of third parties, such as family members or health care workers, during the videoconferencing (VC). In addition, it includes permission for recording audio content or images, and option to request an in-person consultation instead of a digital one.^41^

Required skills for creating a human-oriented relationship comprise two sub-categories: adopting a person-centred approach^32,35,41,43,45^ and building rapport.^34,35,45^ Adopting a person-centred approach includes working with patients to create person-centred options and knowing patients as persons^35^:

They (patients) appreciated being asked questions about their families, occupation, and things that brought them joy and appreciated the nurses remembering these.^35^

It also includes individualised care, that is, tailoring digital approaches to suit individual needs,^32,43^ and considering patients’ language difficulties, hearing impairments^35^ and cultural background.^41^ Building rapport means a connection established with another person based on respect, acceptance, empathy and a mutual engagement.^36^ Required skills for building rapport includes making introductions as needed at the beginning of the appointment,^45^ partnering with the patients to ensure their concerns are responded to,^45^ communicating with patients in a caring way^34^ and using a personal touch during the digital appointment.^35^

Required skills for digital patient education and support comprise three sub-categories: providing information,^31,45^ ensuring understanding and providing support.^45^ Providing information includes setting a common agenda for the appointment, delivering the key information and ending the appointment by summarising the main points and reviewing the next steps.^45^ It also includes information consulting for patients.^31^ Ensuring patients’ understanding includes providing information in a structured way^45^ and encouraging patients to participate.^45,49^ Furthermore, it includes using silence to help patients process information and checking patients’ understanding^45^:

Checking a patient's current understanding of the reason for the visit can help the clinician structure the conversation and fill in knowledge gaps when appropriate.^45^

Providing support includes encouraging expression of patients’ feelings, responding empathically to patient's emotions or experiences and verbally acknowledging support. It also includes acknowledging patient's emotional cues and affecting patient's emotional responses during the digital appointment^45^:

Pay attention to emotional cues from the patient during your visit, such as fidgeting, tuning out, crying, or looking very worried. Verbally acknowledge these cues during the visit.^45^

### Existing digital skills of health care professionals in cancer care

Existing digital skills of HCPs are based on descriptions by HCPs themselves,^31,33^ patients,^38^ or both HCPs and patients.^34,35^ Existing digital skills of HCPs comprise three main categories: skills for (a) *information technology,*^31,33^ (b) creating a human-oriented relationship^34,35,38^ and (c) digital patient education and support^29–31,34,38,40^ (Table 3).

Existing skills for information technology comprise two sub-categories: using digital technologies^31,33^ and assessing digital health information.^31^ Using digital technologies includes work-based computer skills, such as to access online clinical sources and patient outcomes.^33^ It also includes actively searching information from databases for both HCPs and patients.^31^ Assessing digital health information includes assessing information sources and website quality. In addition, it includes filtering relevant information from databases.^31^

Existing skills for creating a human-oriented relationship comprise one sub-category, skills for *building rapport*,^34,35,38^ which includes skills to adapt a bedside manner during VC,^35^ being patient^38^ and using a caring tone of voice and guidance^34^:

Most (patients) felt that providers were very patient during the call, never rushed through questions, and were respectful of scheduled appointment times.^38^

Existing skills for digital patient education and support comprise two sub-categories: HCP–patient interaction,^29–31,34,38^ and providing online support.^40^ HCP–patient interaction includes skills for e-mailing with patients, for example, in relation to laboratory results and prescription renewals.^31^ It also includes communication with care and support.^34^ HCPs reported having skills to express themselves, both effectively^30^ and affectively^29^:

From individual statements (*n* = 28), 19 learners (68%) felt comfortable communicating using telehealth, and 21 learners (75%) indicated that they could express their emotional responses via VMC.^29^

In addition, HCP–patient interaction includes skills to assess patient-reported health information online.^31^ Furthermore, it includes skills for respecting the scheduled appointment times and connecting even at unscheduled times.^38^ Providing online support includes skills for being a facilitator in a digital group chat by acting as a group leader, setting norms, building group cohesion, pacing discussion, using emotional self-disclosure, managing intense emotions and using online tools to enhance group objectives.^40^

### The development areas of digital skills of health care professionals

The development areas of HCPs’ digital skills are based on descriptions by researchers,^28,31,37,39,41,43,44,48^ HCPs themselves,^29,30,32–35,42,46,47,50,51^ or patients.^38,49^ The development areas for digital skills formed five main categories: improving skills for (a) information technology,^33,37,41–43,46,47,51^ (b) ethical practice,^28,30,41,50^ (c) creating a human-oriented relationship,^29,34,38,44,49^ (d) digital patient education and support^28–32,35,36,38,39,41,43,44,48–50^ and (e) implementing digital health (Table 3).^28,34,35,42,49,51^

Developing skills for information technology comprises three sub-categories: improving HCPs’ skills for using digital technologies,^33,41^ accessing digital resources^37,42,43,46,51^ and assessing digital health information.^43,47^ Using digital technologies includes learning basic operational skills for digital equipment, such as video-conferencing,^41^ as well as assessing and developing computer skills^33^:

All physicians and health staff participating in VC and the tele oncology service should undergo formal competency training in communication skills, basic operational skills for VC equipment and cultural awareness.^41^

Accessing digital resources includes assisting patients to navigate digital health^43^ and HCPs adopting new technologies such as artificial intelligence in cancer care.^42,51^ It also includes ways to remind HCPs about updated digital guidelines^37^ and Internet addresses,^47^ as well as enhancing HCPs’ awareness of institutional e-resources.^46^ Assessing digital health information includes providing HCPs information about the content and reliability of cancer-related websites^47^ and guiding their choices as to appropriate and safe digital resources and systems.^43^

Developing skills for ethical practice comprises two sub-categories: protecting patients’ privacy^28,30^ and ensuring confidentiality.^28,30,41,50^ Protecting privacy was a concern in only 6% (*n* = 13) of the HCPs.^30^ Ensuring confidentiality includes accessing patients’ information with their consent,^50^ participation of a third person in a digital appointment^41^ and the confidentiality of conversations^28^:

It was often difficult to have confidential conversations on a telehealth visit conducted in a shared, tight clinic space. Nurses described colleagues’ talking in the background … team members continuously entering and leaving the clinic space while they were trying to focus during a telehealth encounter.^28^

Developing skills for creating a human oriented relationship comprises two sub-categories: building rapport^30,32,34^ and delivering difficult news.^29,44,48^ Building rapport includes developing skills to establish a relationship,^34^ creating an emotional connection with patients^32^ and maintaining a rapport^30^:

… many providers reported difficulties connecting with patients or concerns regarding their ability to forge or maintain a robust provider-patient relationship.^32^

Delivering difficult news comprises two sub-categories: showing support^29^ and responding to patients’ emotions.^44,48^ Showing support includes replacing physical touch and expressing empathy when telling difficult news digitally.^29^ Responding to patients’ emotions includes dealing with patients’ emotional reactions, for example, when they are angry or scared,^48^ responding verbally to patients’ emotional cues, verbalising empathy^44^ and legitimising patients’ concerns^44^:

Future communication skills training programs for telephone-based supportive care needs to focus on responding verbally to patient emotional cues and verballing expressing empathy by health professionals.^44^

Developing skills for digital patient education and support comprises five sub-categories: providing e-counselling,^36^ individualising education,^35,38,43,48,49^ digital interaction,^28,29,34,38,39,41,44,49^ obtaining necessary information^41^ and ensuring patients’ understanding^38,44,48–50^. Improving skills for providing e-counselling is based on the assessment that most HCPs (*n* = 120, 88%) have deficiencies in these skills.^36^ Individualising education focuses on determining what type of appointment will best serve the patient for each encounter^35,38,43,49^ and tailoring information for patients’ characteristics.^48^

Digital interaction includes using different digital media in communication.^29^ It also includes having necessary communication skills^29,41,49^ and keeping nuances during digital communication, as digital appointments are often more factual, shorter and with fewer nuances compared to in-person appointments.^49^ In addition, it includes preserving the human component in interaction,^38^ showing facial expressions,^29,44,49^ and being present with a caring tone of voice and guidance^34^:

Given the significance of empathy and support that patient experience in the cancer centers, attention to preserving the humanistic aspects of cancer care should be at the forefront of this virtual endeavour.^38^

Obtaining necessary information during the digital appointments includes receiving accurate information^39^ as well as reading patients’ reaction to the information provided.^49^ Furthermore, it includes skills to assess patients’ condition^28,38^ and perform physical examinations virtually.^30,32^ Documenting each digital appointment is important.^41^ Ensuring patients’ understanding includes supporting patients to be prepared for the digital appointment,^49^ presenting health information in a structured way, managing information,^48^ and avoiding misinterpretations.^50^ In addition, it includes focusing on interview techniques with open-ended questions,^44^ encouraging patients to ask questions^38,49^ and checking patients’ understanding during digital appointments^44^:

The low number of utterances… and checking patient understanding… confirms the need for skills training to specifically assess clarification of patient understanding.^44^

Developing skills for implementing digital health comprises two sub-categories: creating a standard digital health policy^28,34,49^ and implementing digital health guidelines.^35,42,51^ Creating a standard digital health policy includes updating institution practices on digital health^28^ and defining the factors that constitute a good digital appointment.^34,49^ Skills for implementing digital health guidelines includes ensuring safety on new digital technologies such as artificial intelligence and developing ethical frameworks of new digital technologies to be applied in clinical practice.^51^ In addition, it includes creating frameworks for the development, adoption and regulation of AI technologies in healthcare,^42^ complying with the law^51^ and ensuring that the guidelines are person-centred.^35^

## Discussion

This systematic review produced a synthesis of digital skills of HCPs in cancer care divided into three areas, that is, the required and existing digital skills and the development areas of these skills. Existing and required digital skills of HCPs working in cancer care are a broad skillset covering skills for information technology, ethical practice, creating a human-oriented relationship and digital education and support. The studies were mainly conducted in recent five years, which is understandable considering the topic. According to the findings, the level of HCPs’ existing digital skills was not systematically assessed by the professionals themselves or others involved. Due to the digital transformation in cancer care, there is an emerging need for research on HCPs’ digital skills. Most of the studies showed good internal validity and decent quality. However, they were not specifically designed to study HCPś digital skills but rather, HCPs’ experiences related to digitalisation and its effects on care delivery. Only four studies addressed the digital skills in more detail^33,36,42,46^; however, one of them was designed to evaluate HCPs’ knowledge of institutional e-resources^46^ with fair study quality. Three other studies designed to explore HCPs’ needs to improve their information technology skills,^33^ its current use, perceptions of AI^42^ or training needs for conducting e-counselling^36^ showed lower methodological quality and the results should thus be viewed with caution.

### Required and existing digital skills of HCPs in cancer care

In previous literature, digital skills of HCPs in cancer care were looked at from the perspective of requirements or existing skills. None of the previous studies had compared the required and existing skills or assessed the level of these skills. Both the required and the existing skills had the same main categories that were related to information technology, ethical practice, human-oriented relationship and patient education and support. Some of the content in the main and sub-categories may seem overlapping because we have tried to express their core content in the areas of related content. Next, we discuss their correspondence with earlier studies and digital skills frameworks.^9–11,13^

In information technology, a study related to the required skills for information technology, that is, finding and assessing information from the Internet, was published almost 20 years ago,^31^ and the requirements are likely to be different today. In recent years, requirements in skills related to digital technology have focused on preparing digital appointments, perhaps due to their strong increase in cancer care. HCPs have been required to have basic technical skills to produce digital cancer care services, as also reported in previous studies^12,15,19^ and digital skills frameworks.^11^ In terms of existing skills, HCPs in cancer care perceived having skills to use computers and search and assess online information more than ten years ago.^31,33^ However, a recent study by Jarva et al. (2022) from various healthcare settings reported lack of technical skills related to software or technical equipment.^19^ Since the use of digital technology has increased rapidly in cancer care, technical skills need to be updated accordingly to deliver quality healthcare, as outlined by WHO.^4^ Skills and educational needs can vary considerably between HCPs,^20^ which is why assessment of actual skills and focusing education on deficiencies of these skills is needed.

In ethical practice, the required skills focused on consent to and privacy during digital appointment. This is in line with digital skills’ frameworks related to safe and appropriate information delivery.^12^ Privacy concern is relevant since digital appointments are often held in patients’ home environment, which may compromise privacy. In previous studies, privacy has been related to digital health data within services, such as whom the health information is shared.^52^ Previously, patient consent has been related to users’ right to determine whether or how their personal data is collected, used or shared.^53^ In terms of existing skills, HCPs in cancer care were not concerned with confidentiality and privacy during digital appointments, although the result does not tell us about real skills to protect patients’ privacy. Research on HCPs’ existing digital skills for ethical practice in cancer care is lacking, although ethical values are essential for HCPs.^54^ Therefore, the skills need to be explored further from the perspectives of privacy, confidentiality and consent.

In human-oriented relationship, the required skills were related to a person-centred approach, rapport and including patients’ preference in recently published studies.^32,34,35,43,45^ This is in line with digital skills framework emphasising communication and collaboration in digital skills.^11^ The fear of the patient–HCP relationship deteriorating due to digital technology has been addressed in previous studies,^18,19,52^ indicating that the relationship is seen as uniquely important in cancer care. In individualised care, HCPs need to evaluate whether digital health services are appropriate for the patient's situation^19^; thus, knowing the patient well when making decisions about a digital appointment could enhance individualised cancer care. In terms of existing skills, the only skill reported within human-oriented relationship was building rapport. Some nurses felt that bedside manner can easily be adapted in the digital environment.^35^ It means active listening, connecting with the patients’ story and exploring emotional cues.^55^ Furthermore, patients felt that HCPs had skills to establish a good relationship and communicate in a respectful and supportive way digitally.^34^ Due to lack of physical presence, non-verbal communication through camera may enhance digital communication.^20^ Identifying non-verbal communication and training these skills in the digital environment could support a deeper connection with the patient. In the included studies, skills for individualised, person-centred care were not assessed, although they are essential aspects in the care of people with cancer.

In digital education and support, the required skills entailed ensuring patients’ understanding^45,49^ whereas individual approach in education was not reported as required skills of HCPs. In terms of existing skills, ensuring patients’ understanding was not reported; instead, the skills focused on digital interaction and support. HCPs in cancer care perceived digital interaction to be as easy and effective as in-person interaction, and some of them were able to express themselves emotionally as well.^29,30^ Assessing these skills could benefit the identifying of possible gaps in existing skills in providing support digitally.

### Development areas on digital skills of HCPs in cancer care

Several development areas in HCPs digital skills were identified. Studies on development areas in the skills for information technology were published about ten years ago in Australia and USA,^33,41,47^ meaning that the data need to be updated. The need for skills development continues to focus on technical competence, but the emphasis is more on assessing the reliability of information sources and accessing digital sources such as artificial intelligence globally.^42,51^ Jarva et al.^19^ have stated that using digital health requires skills for finding the correct information but also media literacy to critically evaluate the available information. The need of developing HCPs’ information technology skills has been addressed in previous studies^19,56^ and digital skills framework,^11^ as well.

In ethical practice, the development area was related to privacy and confidentiality during digital appointments. These studies were mostly conducted during the last few years in the USA, Australia and the United Kingdom.^28,30,41,50^ Further research is needed on ethical skills in digital cancer care to ensure its continued use in the future. In creating a human-oriented relationship, the development areas focused on building rapport and expressing empathy. Today, HCPs may lack skills to establish an emotional connection with patients and to maintain rapport digitally.^30,32^ Previously, this has been a challenge from both patients’ and HCPs’ perspective.^19,20,52^ Offering continuing education and opportunities to practice skills for human-oriented relationship digitally could enhance the skills for building rapport in cancer care. In delivering difficult news, the development area was focused on responding patients’ emotions and providing comfort without touching.^29,44,48^ Careful preparation of such a delicate situation and training these skills in clinical settings is needed. HCPs’ skills for delivering difficult news digitally for people with cancer have not been reported in previous studies. This may be connected to HCPs’ preference to deliver difficult news in-person. Continuing education and careful preparation are needed to further develop these skills digitally as in-person appointments are not always possible.

In patient education and support, there were many development areas, the focus being on digital communication skills, which have been studied globally over the years,^41,49,50^ and especially on non-verbal communication skills,^29,44,49^ maybe due to the increasing number of digital appointments in cancer care. In this area, a study reporting lack of skills to conduct e-counselling was topical ten years ago,^36^ which means that findings need to be considered with caution. Studies addressing ensuring patients’ understanding and encouraging patient participation have also been published during the last decade,^29,38,44,49^ indicating global interest in the matter. In digital education and support, the development areas focused on digital interaction and obtaining necessary information. Skills for providing e-counselling was a current area of skills about ten years ago^36^ and it is necessary to update the level of these skills. Studies addressing patients’ understanding and encouraging patient participation have also been published during the last decade, which indicates global interest in the issue.^29,38,44,49^ One development area was assessing patients’ condition remotely, which has been reported in recent years.^28,30,32,38^ Inability to perform a hands-on physical examination digitally requires new skills of HCPs.^18,19^ It could be useful to innovate, together with the patients, how to replace a physical examination at a digital appointment. An important development area is checking patients’ understanding of the information provided during the digital appointment. Given that misunderstandings occur in the digital environment,^50^ practising skills to ensure patients’ understanding could enhance patients’ understanding of the information provided.

In implementing digital health, research conducted in Europe and Australia^42,51^ demonstrates that HCPs can play a significant role in implementing digital services to cancer care practice. Creating a standard policy and introducing guidelines for digital cancer care services were the main development areas in implementing digital health. Those delivering digital cancer care need to be aware of state laws and regulations that govern digital health practice,^18^ especially when implementing new technologies such as artificial intelligence. Legal concerns related to the use of artificial intelligence in cancer care include governance, liability and accuracy.^57^ Digital health ethic codes, on the other hand, focus on the establishment of the principles such as quality, privacy and informed consent among digital services.^17,53^ These recommendations and guidelines are important to implement in cancer care to enhance HCPs’ digital skills.

## Conclusion

This systematic review provides a novel synthesis of the required and existing digital skills of HCPs in cancer care, as well as the development areas of these skills. Both requirements and existing skills were identified in the literature. There are no, however, studies comparing the required and existing skills which could give also future development areas. Therefore, numerous development areas exist, with emphasis on using digital health technology in communication and education and creating an emotional connection with the patient. These skills are needed to ensure successful deployment of digital technologies in cancer care. In clinical practice, continuing education, considering both existing skills of HCPs and clinical requirements, is needed for the further development of these skills. The findings of this review can be used in development activities and research in this field. The required digital skills need clarification related to digital patient education and delivering difficult news. Studies assessing HCPs’ existing digital skills in cancer care are scarce, especially related to ethical practice and patient education. Therefore, quality studies assessing these skills with valid instruments are needed.

### Strengths and limitations

The strength of this review is that it provides a comprehensive synthesis of HCPs’ digital skills in cancer care, identifying both skills that are required, skills that already exist and skills that need to be developed. We also used several databases without time limit, applying along historical perspective. Another strength is that good scientific practice was followed during the research process. The data selection, extraction and analysis process were conducted independently by the authors and confirmed by the research group.^58^

There are also limitations in the review. The first one has to do with the focus of the studies analysed: in the studies, digital skills were mainly described, not assessed. Moreover, in some cases, data was analysed relying on interpretation of the researchers, which might pose a risk to the validity of the results.^25^ No valid tools for assessing the digital skills were identifiable in the literature. The second limitation has to do with the concept of skill, which was described as an independent concept or as part of digital competence, with overlapping between these two concepts. In most of the earlier studies, skill is part of competence, but also an independent concept. Thus, in future studies it would be relevant to clarify this conceptual discrepancy; the problem is, however, that these concepts are not clearly defined in the literature. The third limitation has to do with the nature of the studies included: their methodological quality was not very high, and this clearly limits the generalisation of the results. Thus, the results of this review can be seen as indicating thoughts for future developments.

### Implications for practice, policy and research

The results of the review have several implications. In practice, these implications have to do with existing digital skills. These are skills that professionals can be assumed to have, and practice can rely on them. There are, however, many skills still that require development, which must be considered in clinical practice and in new service fields for patients with cancer, for example, by creating a human-oriented relationship and ensuring patient understanding digitally. To secure high-level ethical practice, issues such as patients’ privacy and confidentiality need to be considered during digital appointments in cancer care. This review indicates the implementation of ethical and practical guidelines to support digital cancer care. Assessment of HCPs’ actual skills and educational needs could further enhance the use of digital technologies in cancer care; thus, development and validation of instruments to assess digital skills of HCPs in cancer care are also implications for future research.

## Supplemental Material

sj-docx-1-dhj-10.1177_20552076241240907 - Supplemental material for Digital skills of health care professionals in cancer care: A systematic review Supplemental material, sj-docx-1-dhj-10.1177_20552076241240907 for Digital skills of health care professionals in cancer care: A systematic review by Tuominen Leena, Poraharju Jenna, Carrion Carme, Lehtiö Leeni, Leino-Kilpi Helena, Moretó Sònia, Stolt Minna, Sulosaari Virpi and Virtanen Heli in DIGITAL HEALTH

sj-docx-2-dhj-10.1177_20552076241240907 - Supplemental material for Digital skills of health care professionals in cancer care: A systematic review Supplemental material, sj-docx-2-dhj-10.1177_20552076241240907 for Digital skills of health care professionals in cancer care: A systematic review by Tuominen Leena, Poraharju Jenna, Carrion Carme, Lehtiö Leeni, Leino-Kilpi Helena, Moretó Sònia, Stolt Minna, Sulosaari Virpi and Virtanen Heli in DIGITAL HEALTH

sj-docx-3-dhj-10.1177_20552076241240907 - Supplemental material for Digital skills of health care professionals in cancer care: A systematic review Supplemental material, sj-docx-3-dhj-10.1177_20552076241240907 for Digital skills of health care professionals in cancer care: A systematic review by Tuominen Leena, Poraharju Jenna, Carrion Carme, Lehtiö Leeni, Leino-Kilpi Helena, Moretó Sònia, Stolt Minna, Sulosaari Virpi and Virtanen Heli in DIGITAL HEALTH
