# Supplementary material for: Digital skills of health care professionals in cancer care: A systematic review
Source: Digit Health. 2024 Mar 24;10:20552076241240907. doi: 10.1177/20552076241240907 (PMC10962045; doi:10.1177/20552076241240907)
Supplement: sj-docx-1-dhj-10.1177_20552076241240907 - Supplemental material for Digital skills of health care professionals in cancer care: A systematic review [file sj-docx-1-dhj-10.1177_20552076241240907.docx]

**Supplementary File 1.** Databases, search strategy, filters and number of results

| Database | Search strategy | Filters | Number of results |
| --- | --- | --- | --- |
| Pubmed | (digital*[tw] OR digitalisation*[tw] OR digitalization*[tw] OR "e-health*"[tw] OR ehealth*[tw] OR mhealth*[tw] OR "Electronic health*"[tw] OR Telecare*[tw] OR "mobile health*"[tw] OR digitisation*[tw] OR digitization*[tw] OR telecommunication*[tw] OR "Telecommunications"[Mesh] OR "tele-based*"[tw] OR "web-based*"[tw] OR "information technolog*"[tw] OR "Information Technology"[Mesh] OR "m-health*"[tw] OR "Digital Technology"[Mesh] OR "Telemedicine"[Mesh] OR telemedicine*[tw] OR telehealth*[tw]) AND ("Professional competence"[Mesh] OR competenc*[tw] OR skill*[tw] OR expertise*[tw] OR "know-how*"[tw] OR capabilit*[tw] OR capacit*[tw] OR knowledge*[tw] OR qualificat*[tw] OR abilit*[tw] OR deficienc*[tw] OR aptitude*[tw] OR proficienc*[tw] OR "Data literac*"[tw]) AND ("Oncology Nursing"[Mesh] OR "Medical Oncology"[Mesh] OR oncolog*[tw] OR "cancer nursing*"[tw] OR "cancer care*"[tw]) NOT ("Review" [Publication Type] OR "Systematic Review"[Publication type] OR "Meta-analysis"[Publication type]) |  | 1178 |
| Cinahl | (MH "Information Technology+" OR MH "Digital Technology+" OR MH "Telecommunications+" OR MH "Telemedicine+" OR MH "Telehealth+" OR MH "Digital Health+" OR digital* OR digitalisation* OR digitalization* OR "e-health*" OR ehealth* OR mhealth* OR "Electronic health*" OR Telecare* OR "mobile health*" OR digitisation* OR digitization* OR telecommunication* OR "tele-based*" OR "web-based*" OR "information technolog*" OR "m-health*" OR telemedicine* OR telehealth*) AND (MH "Professional Competence+" OR competenc* OR skill* OR expertise* OR "know-how*" OR capabilit* OR capacit* OR qualificat* OR abilit* OR knowledge* OR deficienc* OR aptitude* OR proficienc* OR "Data literac*") AND (MH "Oncology+" OR MH "Oncologic Nursing+" OR MH "Oncologic Care+" OR oncolog* OR "cancer nursing*" OR "cancer care*") NOT (PT review* OR PT "meta-analysis" OR PT “meta analysis” OR PT "systematic review") | Peer review | 916 |
| Web of Science Core Collection | Topic: (digital* OR digitalisation* OR digitalization* OR "e-health*" OR ehealth* OR mhealth* OR "Electronic health*" OR Telecare* OR "mobile health*" OR digitisation* OR digitization* OR telecommunication* OR "tele-based*" OR "web-based*" OR "information technolog*" OR "m-health*" OR telemedicine* OR telehealth*) AND (competenc* OR skill* OR expertise* OR "know-how*" OR capabilit* OR capacit* OR qualificat* OR abilit* OR knowledge* OR deficienc* OR aptitude* OR proficienc* OR "Data literac*") AND (oncolog* OR "cancer nursing*" OR "cancer care*") | Excluded: review | 1116 |
| Scopus | (TITLE-ABS(digital* OR digitalisation* OR digitalization* OR "e-health*" OR ehealth* OR mhealth* OR "Electronic health*" OR Telecare* OR "mobile health*" OR digitisation* OR digitization* OR telecommunication* OR "tele-based*" OR "web-based*" OR "information technolog*" OR "m-health*" OR telemedicine* OR telehealth*) OR AUTHKEY(digital* OR digitalisation* OR digitalization* OR "e-health*" OR ehealth* OR mhealth* OR "Electronic health*" OR Telecare* OR "mobile health*" OR digitisation* OR digitization* OR telecommunication* OR "tele-based*" OR "web-based*" OR "information technolog*" OR "m-health*" OR telemedicine* OR telehealth*)) AND (TITLE-ABS(competenc* OR skill* OR expertise* OR "know-how*" OR capabilit* OR capacit* OR qualificat* OR abilit* OR knowledge* OR deficienc* OR aptitude* OR proficienc*) OR AUTHKEY(competenc* OR skill* OR expertise* OR "know-how*" OR capabilit* OR capacit* OR qualificat* OR abilit* OR knowledge* OR deficienc* OR aptitude* OR proficienc*)) AND ( TITLE-ABS(oncolog* OR "cancer nursing*" OR "cancer care*") OR AUTHKEY(oncolog* OR "cancer nursing*" OR "cancer care*")) AND ( EXCLUDE ( DOCTYPE,"re" ) ) AND ( EXCLUDE ( DOCTYPE,"cr" ) ) | Excluded: review and conference review | 1088 |
| Cochrane | (digital* OR digitalisation* OR digitalization* OR "e-health" OR ehealth* OR mhealth* OR Electronic NEXT health* OR Telecare* OR mobile NEXT health* OR digitisation* OR digitization* OR telecommunication* OR "tele-based" OR "web-based" OR information NEXT technolog* OR "m-health" OR telemedicine* OR telehealth*) AND (competenc* OR skill* OR expertise* OR "know-how" OR capabilit* OR capacit* OR qualificat* OR abilit* OR knowledge* OR deficienc* OR aptitude* OR proficienc* OR Data NEXT literac*) AND (oncolog* OR cancer NEXT nursing* OR cancer NEXT care*) | Only Trials | 263 |
| Eric | (digital* OR digitalisation* OR digitalization* OR "e-health*" OR ehealth* OR mhealth* OR "Electronic health*" OR Telecare* OR "mobile health*" OR digitisation* OR digitization* OR telecommunication* OR "tele-based*" OR "web-based*" OR "information technolog*" OR "m-health*" OR telemedicine* OR telehealth*OR DE "Information Technology" OR DE "Web 2.0 Technologies") AND (competenc* OR skill* OR expertise* OR "know-how*" OR capabilit* OR capacit* OR qualificat* OR abilit* OR knowledge* OR deficienc* OR aptitude* OR proficienc* OR "Data literac*" OR DE "Competence") AND (DE "Oncology" OR oncolog* OR "cancer nursing*" OR "cancer care*") | Peer review | 2 |
