# Supplementary material for: Digital skills of health care professionals in cancer care: A systematic review
Source: Digit Health. 2024 Mar 24;10:20552076241240907. doi: 10.1177/20552076241240907 (PMC10962045; doi:10.1177/20552076241240907)
Supplement: sj-docx-2-dhj-10.1177_20552076241240907 - Supplemental material for Digital skills of health care professionals in cancer care: A systematic review [file sj-docx-2-dhj-10.1177_20552076241240907.docx]

**Supplementary File 2.** Quality Appraisal – CASP Checklist for Qualitative Studies ^30^

| Study Id | 1 | 2 | 3 | 4 | 5 | 6 | 7 | 8 | 9 | 10 | Quality rating |
| --- | --- | --- | --- | --- | --- | --- | --- | --- | --- | --- | --- |
| Adames 2023 | Y | Y | Y | Can't tell | Y | Y | Can't tell | Y | Y | Can't tell | **7/10** |
| Bødtcher 2022 | Y | Y | Y | N | Y | Can't tell | Can't tell | Y | Y | Can't tell | **6/10** |
| BORYCKI 2019 | Y | Y | Y | Y | Y | Can't tell | Can't tell | Y | Y | Can't tell | **7/10** |
| Dickerson 2005 | Y | Y | N | N | Y | Can't tell | Can't tell | Y | Can't tell | Can't tell | **4/10** |
| Gotlib Conn 2021 | Y | Y | Y | Can't tell | Y | Can't tell | Can't tell | Y | Y | Can't tell | **6/10** |
| Handley 2020 | Y | Y | Y | Can't tell | Y | Y | Y | Y | Y | Y | **9/10** |
| Jørgensen 2021 | Y | Y | Y | N | Y | Y | Y | Y | Y | Can't tell | **8/10** |
| Karera 2022 | Y | Y | Can't tell | Can't tell | Can't tell | Can't tell | Y | Can't tell | Y | Can't tell | **4/10** |
| Kemp 2020 | Y | Y | Can't tell | Can't tell | Can't tell | Can't tell | Y | Y | Y | Can't tell | **5/10** |
| Koppel 2022 | Y | Y | Y | N | Y | Can't tell | Y | Y | Y | Y | **8/10** |
| Mooi 2012 | Y | Y | N | Y | Can't tell | Can't tell | N | Can't tell | Y | N | **4/10** |
| Stephen 2011 | Y | Y | Y | N | Can't tell | Can't tell | Can't tell | Can't tell | Y | Can't tell | **4/10** |
| Stuij 2018 | Y | Y | Y | N | Y | Can't tell - | Y | Y | Y | Y | **8/10** |

Y: yes, N: no

1. Was there a clear statement of the aims of the research?

2. Is a qualitative methodology appropriate?

3. Was the research design appropriate to address the aims of the research?

4. Was the recruitment strategy appropriate to the aims of the research?

5. Was the data collected in a way that addressed the research issue?

6. Has the relationship between researcher and participants been adequately considered?

7. Have ethical issues been taken into consideration?

8. Was the data analysis sufficiently rigorous?

9. Is there a clear statement of findings?

10. How valuable is the research?
