# Supplementary material for: Digital skills of health care professionals in cancer care: A systematic review
Source: Digit Health. 2024 Mar 24;10:20552076241240907. doi: 10.1177/20552076241240907 (PMC10962045; doi:10.1177/20552076241240907)
Supplement: sj-docx-3-dhj-10.1177_20552076241240907 - Supplemental material for Digital skills of health care professionals in cancer care: A systematic review [file sj-docx-3-dhj-10.1177_20552076241240907.docx]

**Supplementary File 3.** Quality Appraisal – CASP Checklist for Cohort Studies^31^

| Study Id | 1 | 2 | 3 | 4 | 5A | 5B | 6A | 6B | 7 | 8 | 9 | 10 | 11 | 12 | Quality rating |
| --- | --- | --- | --- | --- | --- | --- | --- | --- | --- | --- | --- | --- | --- | --- | --- |
| Damico 2022 | Y | NA | NA | NA | N | N | NA | NA | NA | NA | Y | N | Y | N | **3/9** |
| Hughes 2014 | Y | NA | NA | NA | N | N | NA | NA | NA | NA | Y | N | NA | N | **2/9** |
| Korkmaz 2023 | Y | NA | NA | NA | N | N | NA | NA | NA | NA | Y | N | Y | N | **3/9** |
| Macartney 2012 | Y | Y | NA | NA | N | N | NA | NA | NA | NA | Y | Y | Y | N | **5/9** |
| Maraiki 2018 | Y | Y | NA | Y | N | N | NA | NA | NA | NA | Y | NA | Y | NA | **5/9** |
| Rivet 2023 | Y | NA | NA | N | N | N | NA | NA | NA | NA | Y | N | Y | N | **3/9** |
| Scheetz 2021 | Y | NA | NA | Y | NA | NA | NA | NA | NA | NA | Y | N | Y | N | **3/9** |
| Schnur 2012 | Y | N | - | - | - | - | - | - | - | - | - | - | - | - | **1/9** |
| Shaw 2013 | Y | Y | NA | Y | N | N | NA | NA | NA | NA | Y | NA | Y | NA | **5/9** |
| Emond 2013 | Y | Y | NA | Y | N | N | NA | NA | NA | NA | Y | NA | NA | Y | **5/9** |

Y: yes; N: no; ‘- ‘: when there is ‘N’ for questions 1 and/or 2, the quality assessment is not completed with follow-up questions; ´NA’: the points that don´t fit in the cohort checklist.

1. Are the results of the study valid?

2. Was the cohort recruited in an acceptable way?

3. Was the exposure accurately measured to minimize bias?

4. Was the outcome accurately measured to minimize bias?

5A. Have the authors identified all important confounding factors?

5B. Have the confounding factors been taken account in the design and/or analysis?

6A. Was the follow up of subjects complete enough?

6B. Was the follow up of subjects long enough?

7. What are the results of this study? Box for comments (no possible score)

8. How precise are the results?

9. Do you believe the results?

10. Can the results be applied to the local population?

11. Do the results of this study fit with other available evidence?

12. What are the implications of this study for practice?
